# The Prohibition Issue

**Published:** 1921-09-10

**Authors:** 


### THE PROHIBITION ISSUE.

It is now two years since the great republic of North America "went dry," as the phrase is, and, although no one can contend that similar action is a live issue here, or that Mr. "Pussyfoot" Johnson's mission made any real impression amongst our people, yet it is very distinctly our duty and our business to watch the results of the great experiment in the United States for our own benefit and guidance.

On the one hand, it is asserted that as a result of prohibition, and especially of the "eighteenth amendment" (to the constitution of the United States), and of the Volstead Law, home-brewing, illicit stills, potato-spirit, and other alcoholic beverages are rampant in the land, with the result that those who want alcohol can still get it, but have to pay outrageous prices for very harmful, instead of for the comparatively wholesome and cheaper wines and spirits formerly obtainable. That drugging and other dangerous substitutes for alcoholism are springing up on every hand; that crime is no less, and crimes of violence much more frequent than ever before; that a people dragooned by a law they had no voice in passing reacts by disregarding the law; and that disregard of the prohibition law, so passed, is widespread among every class of citizens.

On the other, it is pointed out that prohibition candidates continue to win at elections; that there are fewer lunatics in the asylums; that public drunkenness is diminishing; that undue prominence is given in the Press to incidents tending to undermine or to throw ridicule on prohibition, for corrupt and pecuniary reasons; that the constant tightening of the laws against possible evasions, as by that which recently forbids doctors to prescribe beer as medicine, is evidence of the general sense that prohibition is good for the country.

*The Times*, in its American number, printed in parallel columns articles for and against prohibition by prominent controversialists, setting forth these and many other arguments. Frankly, the prohibition article seems to us to "cut more ice" than the opposition does. This may be, of course, merely evidence of superior advocacy. Certainly the medical profession is by no means solidly with the prohibitionists. For we note that at the annual convention of the Allied Medical Associations of America the presidential address of Dr. W. W. Fritz declared that the Volstead Law is a curse to the country. Probably the real truth lies partly with both sides of the disputation—namely, that the bulk of respectable men and women who like wine or beer or whisky-and-soda in moderation, and need no protection against themselves from prohibitionists, are now paying through the nose for the privilege of drinking poisonous substitutes for their previous wholesome liquors, but that, on the other hand, the by no means negligible minority who indulged to excess in the old days now find it much more difficult to do so, as well as in many cases beyond their means. It is a question whether total prohibition is the best means of dealing with the drink evil, and whether the much less radical measures taken in this country under stress of war conditions do not, in the end, produce just as much good with few drawbacks and less irritation to the sound and sane bulk of the population. We move more slowly over here, but we have a knack of getting there at the end, and no one can deny that Britain is a much more sober country to-day than it was at the outbreak of war, when it was already more sober than it had ever been at any time of our previous history.

